# A clinical pilot study for personalized risk-based breast cancer screening utilizing a polygenic risk score

**DOI:** 10.1371/journal.pone.0345431

**Published:** 2026-07-08

**Authors:** Tone Hovda, Siim Sober, Peeter Padrik, Krista Kruuv-Kao, Eli Marie Grindedal, Tone Bøe Aaman Vamre, Eline Eikeland, Solveig Hofvind, Kristine Kleivi Sahlberg

**Affiliations:** 1 Vestre Viken Hospital Trust, Drammen, Norway; 2 OÜ Antegenes, Tartu, Estonia; 3 Tartu University Hospital, Tartu, Estonia; 4 Estonian Health Insurance Fund, Tallin, Estonia; 5 Oslo University Hospital, Oslo, Norway; 6 Cancer Registry of Norway, Oslo, Norway; 7 The Arctic University of Norway, Tromsø, Norway; 8 University of Oslo, Oslo, Norway; University of Chicago, UNITED STATES OF AMERICA

## Abstract

**Background:**

Population-based mammographic screening is primarily age-based. However, breast cancer risk is multifactorial, and women may benefit from personalized risk-based screening. This pilot study aimed to explore the use of polygenic risk score (PRS) as a tool for risk stratification in personalized screening.

**Methods:**

We included 80 women aged 40–49 years referred for clinical mammography. Exclusion criteria were prior breast cancer or premalignant breast disease, and previous genetic testing. After DNA collection, PRS was calculated from 2805 Single Nucleotide Polymorphisms (SNPs). Screening recommendations were based on each participant’s relative 10-year breast cancer risk estimated from PRS and compared with the 10-year risk of an average woman of the same age. Women with a self-reported family history of cancer meeting standard criteria were referred for gene panel testing for pathogenic variants in high-risk genes. A follow up questionnaire regarding participants’ experiences was distributed 6–9 months after PRS testing.

**Results:**

Mean age was 45.2 years (SD 2.8). Mean relative 10-year breast cancer risk was 1.18 (SD 0.57). Based on PRS, 40 participants were recommended standard biennial screening 50–69 years, while 40 were advised to begin biennial screening before age 50. Among these, 7 were recommended annual mammography from when their 10-year risk reached twice that of an average 50-year-old. Twenty-one women underwent gene panel testing; no pathogenic variants in breast cancer genes were identified. Five women were advised annual mammography from 40–60 years due to family history of breast cancer, regardless of PRS. Most respondents viewed breast cancer risk assessment positively and did not report increased anxiety after testing.

**Conclusions:**

In this pilot cohort, PRS-based risk stratification led to earlier or more intensive screening recommendations in half of the participants, while family history also influenced management.

## Introduction

Breast cancer is the most common cancer among women in Norway and worldwide [1,2]. Health authorities recommend mammography screening to reduce mortality from the disease through early detection [3,4]. Following European Commission guidelines [3], BreastScreen Norway invites all women aged 50–69 years to biennial mammography screening [5].

Breast cancer development is multifactorial, with risk factors including age, hormonal and reproductive history, mammographic density, lifestyle, environmental exposures, family history of cancer and genetic predisposition [4,6,7]. Despite this complexity, population-based breast cancer screening programs typically apply a “one size fits all” approach, using age and sex as the sole criteria, without addressing individual risk profiles [8–10]. Although mammography screening has proved to reduce breast cancer mortality among participants, challenges persist, such as false positives, overdiagnosis, and diagnosis of advanced-stage cancers or interval cancers diagnosed between screening rounds [11–15]. A more targeted and personalized screening strategy could enhance benefits and reduce harms for the individual women [9,10].

Approximately one third of breast cancer cases are linked to genetic factors [16,17], but only 4–5% are attributable to pathogenic variants in high-risk breast cancer genes, primarily *BRCA1* and *BRCA2* [17]. In Norway, women with a family history of breast or ovarian cancer may be referred for genetic counselling and gene panel testing according to national guidelines [18]. Genome-wide association studies have identified numerous low-risk genetic variations, single nucleotide polymorphisms (SNPs), which collectively explain about one-third of genetic susceptibility [7,17,19–22]. While individual SNPs confer minimal risk, they can be combined into a polygenic risk score (PRS) expressing the total breast cancer risk attributed to these genetic variations. Modeling studies have explored PRS-based stratified screening alone, and combined with other risk factors, such as mammographic density and family history, as well as its integration into existing risk prediction models [7,10,23]. Currently, PRS is not part of clinical prevention guidelines or systematic mammographic screening in Norway.

To inform future prospective studies and to address knowledge gaps, we conducted a clinical pilot study assessing PRS as a tool for personalized breast cancer screening among women aged 40–49 (pre-screening age) in Norway. Our objectives were to explore the use of PRS for risk stratification in mammographic screening, examine its associations with mammographic density, family history, and high-risk gene analyses, and to assess women’s attitudes toward PRS-testing in a screening context.

## Materials and methods

The study was approved by the Norwegian Regional Committee for Medical Research Ethics (REK 494936). All participants provided written informed consent to study participation and use of collected data. The study adhered to the Declaration of Helsinki and relevant regulations. The trial is registered at ClinicalTrials.gov (NCT05731453, registration date 16/02/2023). The authors confirm that all ongoing and related trials for this intervention are registered.

### Study population

**Fig 1:**
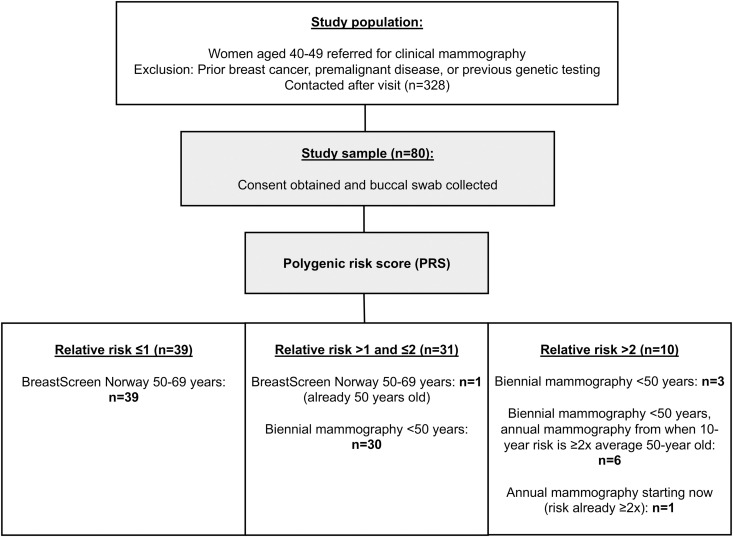
Flow chart.

Women aged 40–49 referred to Vestre Viken breast center for clinical mammography due to breast symptoms between October 1, 2022 and March 31, 2023 were eligible. Exclusion criteria included current or prior breast cancer or premalignant breast disease, or previous genetic testing for familial breast cancer. Eligible women received an SMS after the start of the trial with a link to study information and a digital consent form (Fig 1). Consenting participants completed an online questionnaire on life-style factors and family cancer history. Mammographic density was classified from clinical mammograms using the American College of Radiology’s Breast Imaging – Reporting and Data System (BI-RADS) classification (5^th^ edition), category a-d [24]. Participants were scheduled for DNA sampling at the breast center. As a pilot study, enrollment was limited to 80 women. Data were managed using Ledidi Core (Ledidi AS, Norway).

### Polygenic Risk Score (PRS) and AnteBC test

DNA was collected via Isohelix buccal swabs, stored in Isohelix Buccal Fix tubes (Isohelix, Cell Projects Ltd, UK) and sent to Antegenes (Tartu, Estonia) for PRS analysis using the CE-marked AnteBC test. This test, developed by Antegenes, estimates individualized breast cancer risk using PRS and has been validated in a Norwegian population [22]. It analyzes 2803 breast cancer-associated SNPs to generate an individual PRS value and a 10-year breast cancer risk estimate. Test development was based on large datasets from two population biobanks, the Estonian Biobank (Estonian Genome Center, University of Tartu) and the UK Biobank. Detailed methodological descriptions of AnteBC and its computational framework are presented in Paadrik et.al. [25]. Relative risk for breast cancer was calculated as each participant’s 10-year risk divided by the average 10-year risk for a Norwegian woman of the same age. Based on their relative risks, participants were assigned to one of three risk groups; below average/medium (relative risk ≤1), slightly elevated (relative risk >1 and ≤2) and elevated (relative risk >2).

### PRS-based screening recommendations

As standard practice for starting mammography screening in BreastScreen Norway is at age 50, we defined the “zero point” of risk as the average 10-year breast cancer risk level of 50-year-old women (2.1% based on NORDCAN data [2]). This threshold was used to guide intervention for women with relative risk >1. Some women may never reach this risk level [22], but as no participant was to receive less intensive screening than current standard, all women with relative risk ≤1 were recommended standard screening from age 50–69.

Screening recommendations were tailored to PRS results as follows:

- Relative risk ≤1: Standard biennial mammographic screening at age 50–69 (Breast Screen Norway).
- Relative risk >1 and ≤2: Biennial screening beginning at the age when a woman’s 10-year risk reaches that of an average 50-year-old woman.
- Relative risk >2: Biennial screening from the age when the 10-year risk equals that of an average 50-year-old woman, and/or annual screening once the risk reaches twice that level.

Results and recommendations were communicated by postal letter, with the option of additional consultation if needed.

### Family history of cancer

Participants reported cancer history among first- and second-degree relatives, not restricted to breast or ovarian cancer, using an online questionnaire. Those meeting national criteria for hereditary cancer testing [18] were referred to the Section for Hereditary Cancer at the Oslo University Hospital for genetic counselling and testing using a standard 29-gene panel *(APC, ATM, BAP1, BMPR1A, BRCA1, BRCA2, BRIP1, CDKN2A, CDK4, CHEK2, EPCAM, FLCN, HOXB13, MLH1, MSH2, MSH6, MUTYH, PALB2, PMS2, POLE, POLD1, PTEN, RAD51C, RAD51D, SDHB, SMAD4, STK11, TP53* and *VHL)*. Women with negative results but meeting guideline criteria (Table 1) were advised annual mammography from age 40–60 [18]. In case of conflicting recommendations, the most intensive schedule was advised.

**Table 1:** Criteria for annual mammography from ages 40-60 in women without pathogenic variants in high-risk breast cancer genes.

Annual mammography is recommended when any of the following criteria are met:
• One first-degree relative diagnosed with breast cancer at ≤40 years*
• Two-first degree relatives with breast cancer, with a mean age at onset ≤55 years
• Three close relatives with breast cancer, with a mean age at onset ≤60 years
• Four close relatives with breast cancer, regardless of age at onset

*If the affected relative was < 40 years at diagnosis, screening is recommended from age 30.

### Follow-up

A digital questionnaire was sent to all participants 6–9 months post-inclusion to assess their experiences (S1 Table). Responses were recorded on a five-point scale and collapsed into three categories: disagree, neutral, and agree.

### Statistical analyses

Descriptive statistics were used. Age, PRS-values, and relative 10-year risk were summarized as means with standard deviations (SD) and medians with interquartile ranges (IQR). Relative risk was categorized (≤1, > 1 and ≤2, > 2; ≤ 1.5 and>1.5). Analyses were stratified by mammographic density (low – BI-RADS a or b; high – BI-RADS c or d), genetic testing eligibility, and screening recommendations based on family history. Group differences were tested using two-sample t-tests, chi-square tests, or Fisher’s exact tests; p < 0.05 was considered statistically significant. To account for multiple testing, Bonferroni correction was applied separately for each set of related analyses. Results from the follow-up questionnaire about women’s experiences were presented as proportions (disagree, neutral, agree) for all and stratified by relative risk (≤1 or >1). All analyses were performed in IBM SPSS Statistics v.29.

## Results

A total of 320 eligible women were invited to achieve the target sample size of 80, as specified in the study protocol. This resulted in a 25% response rate (80/320). The mean age of participants was 45.2 years (SD 2.8), and the median age was 45.0 years (IQR 43.0–47.5) (Table 2).

**Table 2:** Age, family history of cancer, PRS-value, and relative PRS-based breast cancer risk stratified by mammographic density.

	Total	BI-RADS a/b	BI-RADS c/d	p-value*
N (%)	80 (100%)	41 (51%)	39 (49%)	
**Age (years)**				
Mean (SD)	45.2 (2.8)	44.4 (2.9)	46.0 (2.6)	0.009
Median (IQR)	45.0 (43.0-47.5)	44.0 (42.0-47.0)	47.0 (44.0-48.0)	
**Family cancer history**				
Positive	25 (31%)	12 (29%)	13 (33%)	0.70
Negative	55 (69%)	29 (71%)	26 (67%)	
**PRS-value**				
Mean (SD)	0.13 (1.09)	0.23 (1.01)	0.02 (1.17)	0.38
Median (IQR)	0.15 (−0.52-0.88)	0.34 (−0.44-0.88)	−0.15 (−0.79-0.60)	
**Relative risk**				
Mean (SD)	1.18 (0.57)	1.22 (0.53)	1.14 (0.61)	0.58
Median (IQR)	1.07 (0.80-1.48)	1.16 (0.82-1.48)	0.94 (0.71-1.30)	
**Relative risk**				
≤ 1	39 (49%)	18 (44%)	21 (54%)	0.34
> 1 and ≤2	31 (39%)	19 (46%)	12 (31%)	
> 2	10 (12%)	4 (10%)	6 (15%)	
**Relative risk**				
≤ 1.5	61 (76%)	31 (76%)	30 (77%)	0.89
> 1.5	19 (24%)	10 (24%)	9 (23%)	

* After Bonferroni correction (n=6), p<0.008 is considered statistically significant.

### Polygenic risk score and screening recommendations

The mean PRS value was 0.13 (SD 1.09) and the median PRS value was 0.15 (IQR −0.52–0.88). Mean relative 10-year breast cancer risk based on PRS for participants compared to age-matched population was 1.18 (SD 0.57), median 1.07 (IQR 0.80–1.48) (Table 2).

Among the 80 women included in the study, 49% (39/80) had a relative breast cancer risk of 1 or lower based on their PRS. Thirty-nine percent (31/80) had a relative risk between 1 and 2, while 12% (10/80) had a relative risk greater than 2 (Table 2).

Based on these results, 50% (40/80) were advised standard biennial mammographic screening at ages 50–69. Further, 49% (39/80) were advised to start biennial screening before age 50, at the age when their 10-year risk equaled that of an average 50-year-old. Among these, six women (15%) were further recommended annual screening once their risk doubled that of an average 50-year-old. One of the 80 participants (1%) was advised to begin annual screening immediately because her risk was already at that level (Fig 1).

When dichotomized at an alternative threshold of 1.5, 76% (61/80) were classified as low risk (≤1.5) and 24% (19/80) as high risk (>1.5) (Table 2), although this stratification was not linked to separate recommendations.

Regarding breast density, 51% (41/80) were categorized as BI-RADS a or b (low density), and 49% (39/80) as BI-RADS c or d (high density). No statistically significant association was observed between PRS and mammographic density (Table 2).

### Family history and genetic testing

Based on family history, 29% (23/80) were referred for genetic counselling and testing with a 29-gene-panel; 70% (16/23) had a family history of breast or ovarian cancer. Two participants did not provide samples, leaving 21 tested participants. No pathogenic variants in breast cancer genes were identified, although one participant carried a *CDKN2A* variant associated with melanoma and pancreatic cancer risk. Five women (24%) met criteria for annual mammography from age 40–60 based on family history (Table 1) [18].

Mean PRS among gene panel tested participants was 0.45 (SD 1.01) versus 0.03 (SD 1.10) for non-tested (p = 0.13). Median PRS-values were 0.39 (IQR −0.31–1.18) for tested and −0.05 (IQR −0.69–0.73) for non-tested (Table 3). The proportions of tested women with a relative breast cancer risk >1.5 was 43% (9/21) for tested compared with 18% (10/57) for non-tested. The association was nominally significant (p = 0.021) but did not withstand the Bonferroni correction for multiple testing (Table 3). For the five women recommended annual screening from age 40–60 based on family history, mean PRS was 1.39 (SD 0.30) compared with 0.15 (SD 0.99) for others (p = 0.018). Further, all five also had relative risk >1, reinforcing intensive screening recommendations (Table 3).

**Table 3:** PRS value and relative risk stratified by family history of cancer.

Genetic counselling and gene panel testing due to positive family history of cancer*			
	Yes	No	p-value**
**N (%)**	21 (27%)	57 (73%)	
**PRS-value**			
Mean (SD)	0.45 (1.01)	0.03 (1.10)	0.13
Median (IQR)	0.39 (−0.31-1.18)	−0.05 (−0.69-0.73)	
**Relative risk**			
≤ 1	8 (38%)	29 (51%)	0.60
> 1 and ≤2	10 (48%)	21 (37%)	
> 2	3 (14%)	7 (12%)	
**Relative risk**			
≤ 1.5	12 (57%)	47 (82%)	0.021
> 1.5	9 (43%)	10 (18%)	
**Recommended annual mammography screening based on family history of cancer**			
	**Yes**	**No**	**p-value**
**N (%)**	5 (24%)	16 (76%)	
**PRS-value**			
Mean (SD)	1.39 (0.68)	0.15 (0.99)	0.018
Median (IQR)	1.55 (1.18-1.73)	0.09 (−0.45-1.02)	
**Relative risk**			
≤ 1	–	8 (50%)	0.059
> 1 and ≤2	3 (60%)	7 (44%)	
> 2	2 (40%)	1 (6%)	

*Data not available: n = 2.

** After Bonferroni correction (n = 5), p < 0.01 is considered statistically significant.

### Women’s experiences

Of the 80 participants, 73% (58/80) responded to the follow-up questionnaire. Among respondents, 43% (25/58) had a PRS-based relative risk ≤1 and 57% (33/58) had a relative risk >1. In total, 24% (14/58) of the respondents were referred for gene panel testing due to family history.

**Fig 2:**
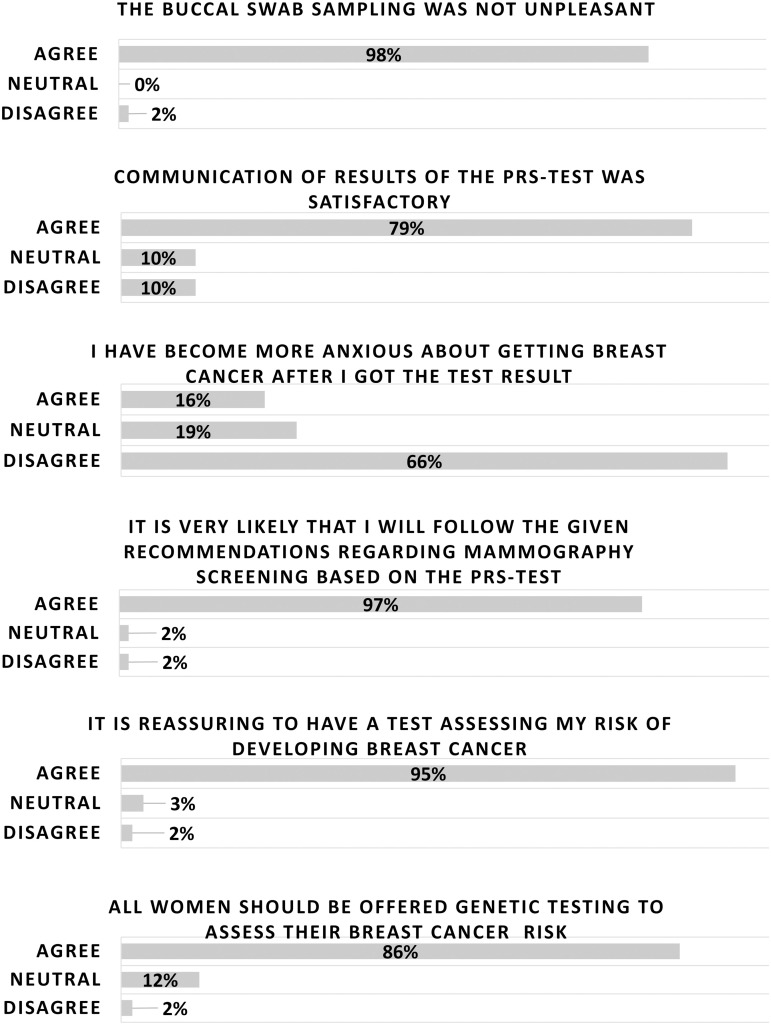
Women’s experiences with participation in the study. Total number of respondents: 58.

**Fig 3:**
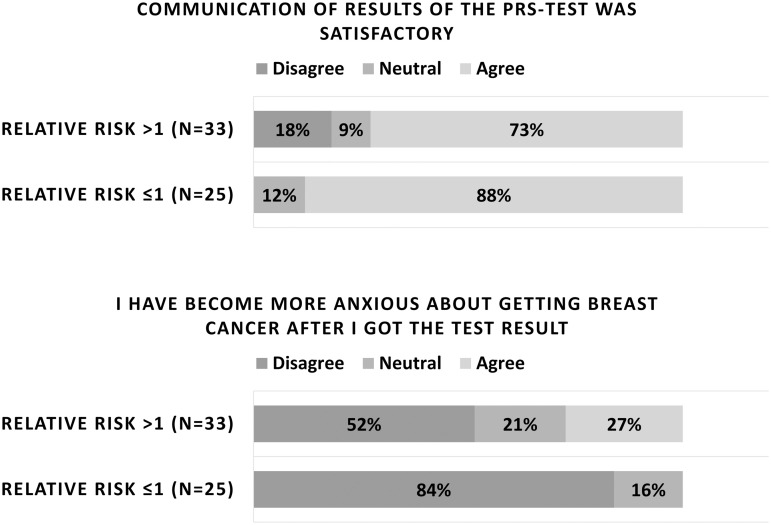
Women’s views on communication of results and anxiety related to breast cancer. Data are stratified by PRS-based relative risk (≤1 vs. > 1). Total number of respondents: 58.

Most women (98% (57/58)) found buccal swab sampling acceptable (Fig 2). Communication of PRS results was considered satisfactory by 79% (46/58), while 10% (6/58) were neutral. Another 10% (6/58) were dissatisfied, all of whom had a relative risk >1 (Fig 3), and they expressed a preference for phone communication rather than letters. Four women with increased risk contacted the breast center for additional information.

Regarding anxiety, 66% (38/58) of respondents disagreed that testing made them more anxious, 19% (11/58) were neutral, and 16% (9/58) agreed (Fig 2). All the respondents who were neutral or who reported increased anxiety had a relative risk >1 (Fig 3).

Almost all respondents, 97% (56/58), intended to follow the screening recommendations given, and 95% (55/58) found testing reassuring. Furthermore, 86% (50/58) agreed that all women should be offered genetic testing to assess breast cancer risk (Fig 2).

**Fig 4:**
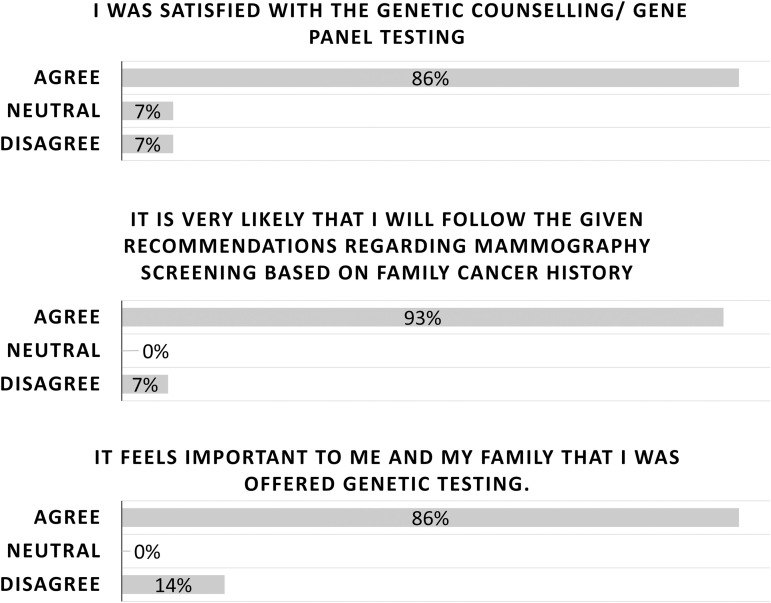
Women’s experiences following referral to genetic counselling and gene panel testing due to family cancer history. Total number of respondents: 14.

Among the 14 respondents referred to genetic counselling and gene panel testing due to family history, 86% (12/14) were satisfied with the process, 93% (13/14) intended to follow recommendations, and 86% (12/14) considered testing important for themselves and their families (Fig 4).

Respondents’ detailed answers to all questions are provided in S2 Table.

## Discussion

We conducted a prospective clinical pilot study including 80 women aged 40–49 years with no prior history of breast cancer or premalignant breast disease. We analyzed each participant’s polygenic risk score (PRS) and explored their experiences with study participation. Half of the women had a PRS-based 10-year breast cancer risk higher than average for women of the same age and were therefore advised earlier or more intensive screening than standard biennial screening. Based on self-reported family history, 27% underwent genetic counselling and testing for pathogenic variants in high-risk cancer genes in accordance with national guidelines. Participants were generally satisfied with participation and expressed positive attitudes toward individualized breast cancer risk assessment. A minority reported increased breast cancer-related anxiety after receiving their test results, all of whom had an elevated PRS-based risk.

In BreastScreen Norway, women receive their first screening invitation at age 50 (more precisely between ages 48–52, depending on residence and the biennial screening interval), and each woman receives at total of ten invitations [5]. However, as our results demonstrate, some women reach the breast cancer risk level of an average 50-year-old at considerably younger age and may therefore benefit from an earlier onset of mammographic screening. This pattern has also been shown by Akdeniz et al., who analyzed Norwegian data using the AnteBC 2803-SNP test. In their study, women in the 99^th^ PRS percentile had a lifetime cumulative breast cancer risk nearly six times higher than women in the 1^st^ percentile, whereas women in the lowest percentiles never reached the average risk of a 50-year-old woman [22].

Polygenic risk scores have shown strong value in risk-stratification models, particularly when combined with other established risk factors such as breast density and family history [17,26,27]. We did not observe a statistically significant association between PRS and breast density. These findings are consistent with previous research showing that PRS and breast density independently influence breast cancer risk without being correlated [28,29]. Breast cancer risk is estimated to be 4–6 times higher in women with extremely dense breasts compared to those with predominantly fatty breasts [30]. However, breast density is not routinely used for risk assessment or risk stratification in Norway, either within the national screening program or in clinical mammography practice.

Among women referred for gene panel testing, a higher proportion had a relative 10-year breast cancer risk >1.5 compared with those not referred. Similarly, women recommended more intensive screening due to family history of breast cancer had a higher mean PRS value.

Although these associations reached nominal statistical significance, they did not remain significant after Bonferroni correction, likely reflecting limited statistical power due to the small sample size and multiple comparisons. These findings may nevertheless suggest a potential underlying association, and support results from larger studies showing that common genetic variations (SNPs) contribute meaningfully to the hereditary component of breast cancer.

Two large ongoing trials, My Personalized Breast Screening (MyPebs) trial [31] and the Women Informed to Screen Depending on Measures of Risk (WISDOM) trial [8] are currently evaluating whether risk-based screening is non-inferior to standard age-based screening with respect to rates of stage II cancers. Their results will provide essential evidence to inform future screening strategies. Implementation of personalized screening programs also requires careful attention to logistics, particularly when genetic testing is involved. In our study, all participants attended an appointment at the breast center for buccal swab sampling. For large-scale implementation, more efficient strategies such as mailing self-collection kits and using digital platforms for communicating test results and screening recommendations would be necessary.

Intensified screening of a high-risk subset of women will inevitably increase workload and costs for the health care system, underscoring the need for careful evaluation of risk thresholds for intervention. In this clinical pilot, we used a relative risk >1 to recommend earlier start of mammographic screening, resulting in half of the participants being advised to start screening before age 50. Raising the threshold to relative risk >1.5 reduced the proportion in the increased-risk group to 24%. Incorporating PRS into risk assessment models including other risk factors can modify the risk assessment for individual women and may influence the number of additional screening examinations required. Risk prediction models such as the Breast and Ovarian Analysis of Disease Incidence and Carrier Estimation Algorithm (BOADICEA)/CanRisk are valuable tools in this context [7,32], but were not applied in this study as they are not routinely used in Norway.

In the context of implementing PRS in personalized screening, it is anticipated that not all women will consent to genetic testing for breast cancer risk assessment. This may challenge principles of equality if women who decline testing are assigned screening pathways perceived as suboptimal. It is therefore essential that screening offered to women who do not consent to genetic testing remains at least equivalent to the current standard. In our study, participation was 25% among the 320 women invited, suggesting a degree of reluctance toward genetic testing and risk assessment in the general population. Although most respondents expressed support for offering all women an assessment of their genetic breast cancer risk, this finding is biased by the fact that all respondents had already agreed to take part in a study with genetic testing as a core component. As one participant reflected: *“I was not sure if it was a good idea to test. I was worried to know if the increased risk would hurt me more than help.”* Among respondents with increased risk based on PRS, approximately one in four reported heightened anxiety about breast cancer following participation, meaning three in four did not. Some degree of anxiety is an expected and reasonable response to the disclosure of elevated risk. Structured communication and follow-up for these women are therefore important, extending beyond the provision of screening recommendations alone.. Understanding the impact of positive test results on women’s quality of life, including short- and long-term psychological responses, remains a key area for future research.

The most prominent limitation of our study was the sample size of only 80 participants, which limits statistical power. However, as a pilot study, the primary objective was to inform the design and feasibility of a future, larger prospective study. The study population, consisting of women referred for clinical mammography, may not be representative of the general population of women in their pre-screening age, limiting external generalizability. In addition, exclusion of women with prior breast cancer and those already referred for genetic testing may have resulted in a lower-risk cohort. Selection bias was likely, as participation required an active choice both regarding enrolment and the follow-up survey, favoring women with more positive attitudes toward genetic testing or risk-stratified screening. This consideration is also relevant for future implementation, as PRS-based screening would be voluntary. Finally, some of the questions in the follow-up questionnaire may have been perceived as leading; however, the consistency of responses suggests that this is unlikely to have substantially influenced the overall findings.

## Conclusions

In this pilot cohort, PRS-based risk stratification led to earlier or more intensive screening recommendations in half of the participants. Family history may add value to risk stratification. Participants were generally satisfied with their involvement and expressed positive attitudes toward individualized breast cancer risk assessment.

## Supporting information

S1 Table Follow-up questionnaire sent to all participants 6–9 months after PRS-testing. Response was given for each question on a five-point scale: Completely disagree, slightly disagree, neutral, slightly agree and totally agree.
(DOCX)

S2 Table Participants’ answers to all questions in the follow-up questionnaire as listed in S1 Table. Response given on a five-point scale. Total respondents question no. 1–8: n = 58. Total respondents question no. 9–13: n = 14.
(DOCX)

S1 File Trial Protocol. (PDF)

S2 File TREND checklist. (PDF)
