# Supplementary material for: A clinical pilot study for personalized risk-based breast cancer screening utilizing a polygenic risk score
Source: PLoS One. 2026 Jul 8;21(7):e0345431. doi: 10.1371/journal.pone.0345431 (PMC13345395; doi:10.1371/journal.pone.0345431)
Supplement: S1 Table — Response was given for each question on a five-point scale: Completely disagree, slightly disagree, neutral, slightly agree and totally agree. (DOCX) [file pone.0345431.s001.docx]

**Supporting information 1**

**S1 Table. Follow-up questionnaire sent to all participants 6-9 months after PRS-testing.** Response was given for each question on a five-point scale: Completely disagree, slightly disagree, neutral, slightly agree and totally agree.

| **To all participants** |
| --- |
| 1. Information given in the invitation to the study was sufficient and satisfactory |
| 2. The buccal swab sampling was not unpleasant |
| 3. The waiting time for the results of the PRS-test was long |
| 4. Communication of results of the PRS-test was satisfactory |
| 5. It is very likely that I will follow the recommendations given to me regarding further |
| follow-up with mammography based on the PRS-test |
| 6. I have become more anxious about getting breast cancer after I got the test result |
| 7. It feels reassuring that I have had a test carried out informing me about my risk of |
| developing breast cancer |
| 8. I think all women should be offered genetic testing to assess their breast cancer risk |
| **To participants referred for extended genetic testing for monogenic pathogenic variants** |
| 9. It was reassuring to be referred for an extended genetic testing |
| 10. I was satisfied with the extended counselling/testing |
| 11. It is very likely that I will follow the recommendations given to me regarding further |
| follow-up with mammography based on the extended testing |
| 12. It feels important to my family and me that I was offered genetic testing. |
