# Supplementary material for: A clinical pilot study for personalized risk-based breast cancer screening utilizing a polygenic risk score
Source: PLoS One. 2026 Jul 8;21(7):e0345431. doi: 10.1371/journal.pone.0345431 (PMC13345395; doi:10.1371/journal.pone.0345431)
Supplement: S2 Table — Response given on a five-point scale. Total respondents question no. 1–8: n = 58. Total respondents question no. 9–13: n = 14. (DOCX) [file pone.0345431.s002.docx]

**Supporting information 2**

**S2 Table. Participants’ answers to all questions in the follow-up questionnaire as listed in S1 Table.** Response given on a five-point scale. Total respondents question no. 1-8: n=58. Total respondents question no. 9-13: n=14.

| **Question no.** | **Completely disagree** | **Slightly disagree** | **Neutral** | **Somewhat agree** | **Totally agree** |  |
| --- | --- | --- | --- | --- | --- | --- |
|  |  |  |  |  |  |  |
| **1** | 1 (2%) | 2 (3%) | 3 (5%) | 8 (14%) | 44 (76%) |  |
| **2** | 1 (2%) | . | . | 3 (5%) | 54 (93%) |  |
| **3** | 8 (14%) | 8 (14%) | 17 (29%) | 22 (38%) | 3 (5%) |  |
| **4** | 2 (3%) | 4 (7%) | 6 (10%) | 9 (16%) | 37 (64%) |  |
| **5** | 1 (2%) | 1 (2%) | . | 4 (7%) | 52 (90%) |  |
| **6** | 29 (50%) | 9 (16%) | 11 (19%) | 8 (14%) | 1 (2%) |  |
| **7** | . | 1 (2%) | 2 (3%) | 14 (24%) | 41 (71%) |  |
| **8** | . | 1 (2%) | 7 (12%) | 6 (10%) | 44 (76%) |  |
| **9** | 1(7%) | . | 1(7%) | 2(14%) | 10(71%) |  |
| **10** | 1(7%) | . | 1(7%) | 2(14%) | 10(71%) |  |
| **11** | 5 (36%) | . | 2(14%) | 6 (43%) | 1 (7%) |  |
| **12** | 1(7%) | . | . | 1(7%) | 12 (86%) |  |
| **13** | 1(7%) | 1(7%) | . | 2(14%) | 10(71%) |  |
