# Supplementary material for: A clinical pilot study for personalized risk-based breast cancer screening utilizing a polygenic risk score
Source: PLoS One. 2026 Jul 8;21(7):e0345431. doi: 10.1371/journal.pone.0345431 (PMC13345395; doi:10.1371/journal.pone.0345431)
Supplement: S1 File — (PDF) [file pone.0345431.s003.pdf]

Study Title:

IMPLEMENTATION OF A MODEL FOR PERSONALIZED RISK-BASED BREAST CANCER SCREENING

Internal Reference Number / Short title: AnteNOR

Ethics Ref:

Date and Version No: March 2023, Ver 02.

**Principal Investigator: Tone Hovda, MD, PhD**<br><br>Position: Breast radiologist, post-doc candidate<br><br>Institution: Vestre Viken HF
**Investigators:**<br><br>**Kristine Kleivi Sahlberg, MSc, PhD**<br><br>Position: Head of research and innovation<br><br>Institution: Vestre Viken HF<br><br>**Helle Skjerven, MD**<br><br>Position: Head of Breast- and endocrine surgery<br><br>Institution: Vestre Viken HF<br><br>**Lovise Mæle, MD, PhD**<br><br>Position: Head of Section for inheritable cancer<br><br>Institution: Oslo University Hospital<br><br>**Eli Grindedal, PhD**<br><br>Position: Genetic Counsellor<br><br>Institution: Oslo University Hospital<br><br>**Vessela Kristensen, MD, PhD**<br><br>Position: Professor<br><br>Institution: Oslo University Hospital<br><br>**Linda Romundstad, MD**<br><br>Position: Head of Breast Diagnostic Centre<br><br>Institution: Vestre Viken HF

**Solveig Hofvind; PhD**<br>Position: Head of BreastScreen Norway<br>Institution: Cancer Registry of Norway<br>**Peeter Padrik, MD, PhD**<br>Position: Oncologist<br>Institution: OÜ Antegenes<br>**Krista Kruuv-Käo, MD**<br>Position: Study Coordinator<br>Institution: OÜ Antegenes<br>**Siim Sõber, PhD**<br>Position: Geneticist<br>Institution: OÜ Antegenes<br>**Regina Saar**<br>Position: Bioinformatician<br>Institution: OÜ Antegenes<br>**Anette Paas**<br>Position: Study Nurse<br>Institution: OÜ Antegenes	
**Sponsor:**	AnteNOR Consortium
**Funder:**	A grant from Enterprise Estonia from the program funded by the Norwegian Financial Mechanism: **Estonian – Norwegian** c**ooperation program "Green ICT"**

Confidentiality Statement

This document contains confidential information that must not be disclosed to anyone other than the Sponsor, the Investigator Team, and members of the Research Ethics Committee unless authorized to do so.

TABLE OF CONTENTS

1. SYNOPSIS 5
2. ABBREVIATIONS 6
3. BACKGROUND AND RATIONALE 6
3.1. BC as a major health problem globally and in Norway 6
3.2. Current BC population-based screening in Norway 7
3.3. The need for personalized risk-based screening 7
3.4. Tools for BC risk prediction 8
Breast density (BD) 8
Genetic predisposition 9
BC risk prediction models 12
3.5. BC Risk Categories 13
3.6. Cost-effectiveness data of personalized risk-based screening 14
3.7. Background Summary and Study Rationale 15
4. OBJECTIVES AND OUTCOME MEASURES 16
5. STUDY DESIGN 18
5.1. Study Participants 18
Inclusion Criteria: 18
Exclusion Criteria: 18
Research methodology 18
5.2. STUDY PROCEDURES AND RECRUITMENT 18
5.3. BLODD SAMPLES/BIOBANK 21
5.4. ASSESSMENTS 21
5.5. Discontinuation/Withdrawal of Participants from Study 23
5.6. Definition of End of Study 23
6. STATISTICAL METHODS 23
6.1. Description of Statistical Methods 23
6.2. Analysis of Outcome Measures 23
6.3. Access to Data 25
7. QUALITY ASSURANCE PROCEDURES 25
8. ETHICAL AND REGULATORY CONSIDERATIONS 25
8.1. Declaration of Helsinki 25
8.2. Guidelines for Good Clinical Practice 25

8.3. Approvals 25
8.4. Reporting 25
8.5. Participant Confidentiality 25
8.6. Expenses and Benefits 25
9. FINANCE AND INSURANCE 25
9.1. Funding 26
9.2. Insurance 26
10. PUBLICATION POLICY 26
11. APPENDIX A: AMENDMENT HISTORY 26
12. REFERENCES 27

1. SYNOPSIS

Study Title	**A CLINICAL PILOT STUDY FOR PERSONALIZED RISK-BASED BREAST CANCER SCREENING**	
**Internal ref. no. / short title**	AnteNOR	
**Study Design**	A pilot cohort study	
**Study Participants**	Healthy women at age 40 - 49	
**Planned Sample Size**	80	
**Planned Study Period**	01.11.2022 – 01.11.2035	
	**Objectives**	**Outcome Measures**
**Primary**	To evaluate the impact of implementing population-based genetics testing strategy for breast cancer precision prevention using the polygenic risk score and monogenic pathogenic variant (MPV) testing in the Norwegian healthcare setting.	Elevated-risk breast cancer polygenic risk score and monogenic pathogenic variants prevalence for precision screening - the proportion of women with elevated risk for personalized screening
**Secondary**	To evaluate the feasibility, potential clinical utility, and cost-effectiveness of a population-based genetic testing strategy for BC precision prevention.<br><br>A scientific study testing the relationship between polygenic risk scores and clinical risk factors, such as mammographic density.	• Clinical utility and effectiveness of the genetics-based personalized precision prevention.<br>• Cost-effectiveness of the genetics-based personalized precision prevention.<br>• Feasibility of the genetics-based personalized precision prevention.<br>• Acceptability, values, preferences, and social norms for personalized precision prevention.<br>• Equitable accessibility of genetics-based personalized precision prevention.<br>• Health-care system readiness for the implementation of personalized precision prevention.<br>• Results of high and moderate risk genetic

		testing (BRCA1, BRCA2, TP53, STK11, PTEN, CDH1, ATM, PALB2, CHEK2, NBN, NF1).<br>• Results of SNPs testing and SNP PRS analyses.<br>• PRS risk versus breast density.<br>• Individuals' distribution into risk groups.<br>• Individuals' participation rate from additional counselling; analysis of patients' compliance.<br>• Exact analysis and description of medical activities after counselling.<br>• The number of family members invited to additional counselling and their participation rate.<br>• Costs analysis of all activities.

2. ABBREVIATIONS

BC – breast cancer

PRS – polygenic risk score

SNP – single nucleotide polymorphism

BD – breast density

MD – mammographic density

3. BACKGROUND AND RATIONALE

3.1. BC as a major health problem globally and in Norway

Breast cancer (BC) is the leading cause of cancer deaths in women. Every year adds 2.3 million new diagnoses and 685 000 deaths (1). BC continues also to be an important public health issue in Norway. BC has been the most common cancer in women also in Norway. There has been a monotonous increase in the rate up to the 1990s with a steeper increase in the mid-1990s followed by a slight decline between

2005 and 2009. There were 650 deaths from BC in 2018, age-standardized (Norwegian standard) mortality rate per 100 000 person-years was 22.0.

BC cases by age groups in 2015-2019:

Age 25-49 years: 3428, that is 686 cases per year (19,4%).

Age 50-69 years: 9201, that is 1840 cases per year (51,9%).

Age 70+ years: 5083, that is 1017 cases per year (28,7%).

According to Globocan data projections BC constitutes in Norway 22.9% of all cancer cases among women with an age-standardized (World) incidence rate of 83.1 per 100,000 and mortality rate of 10.7 per 100, 000 (2).

Measures to avoid premature deaths from BC should combine prevention, early detection, and improved treatment. Our project focuses on better prevention and early detection of BC.

3.2. Current BC population-based screening in Norway

BC screening is widely implemented in many healthcare systems to reduce BC mortality through the earlier diagnosis of smaller, asymptomatic breast cancers. Screening with mammography reduces BC mortality risk 20-40% (3-5). The Norwegian BC screening program Breast Screen Norway started in 1996 and became nationwide in 2005. It invites women aged 50-69 years to biennial mammographic screening.

It is estimated that in Norway screening has reduced BC mortality by 20% (HR = 0.80, 95% confidence interval [CI] = 0.70 to 0.91) among women 50 years and older and by 25% (HR = 0.75, 95% CI = 0.65 to 0.86) among screening-aged women (6). The treatment effect has been 23% (HR = 0.77, 95% CI = 0.65 to 0.92) for women 50 years and older and 17% (HR = 0.83, 95% CI = 0.74 to 0.94) for screening-aged women (6).

We can conclude that currently in Norway BC screening mammography is an accepted and publicly funded early BC detection measure for women in the age group 50-69, where the average BC 10-year risk at age 50 is 2.1% using NORDCAN data (https://www-dep.iarc.fr/NORDCAN/english/frame.asp) and iCARE absolute risk calculation (7).

3.3. The need for personalized risk-based screening

Current BC screening guidelines are primarily based on age and do not support regular screening of women below the age of 50. This largely excludes young women at high risk for developing BC. On the other side, mammography screening has also potential harms and costs, therefore it is not reasonable to implement screening for all women under the age of 50. It is no longer appropriate to apply a single recommendation to women across all age groups and risk profiles and a far more precise and personalized risk estimation is needed. Screening with mammography reduces BC mortality risk 20-40% (3-5). The decline varies across age groups, ranging from 8% in the age group 39-49 years to 33% in the age group 60-69 years. The largest decrease in mortality has been shown in the age group 50-69 years, the evidence base for efficacy is weaker in younger women. The relationship between benefits and potential harms for screening in the age group 40-50 years has been controversial and in most countries

screening is not offered in this risk group. Reduced BC deaths may come at the expense of overdiagnosis (the identification and unnecessary treatment of clinically insignificant tumors), as well as the consequences of false-positive or false-negative results (5, 8).

An age-based population-level breast screening strategy that reduces BC mortality but does not account for the wide variation in individual women's cancer risks. A woman's risk of developing BC is influenced by many factors, but BC screening recommendations are based primarily on age. There are disagreements in the medical community about the optimal age to begin BC screening, as well as the frequency of screening and the age at which to stop. In Norway and mostly globally, the current BC screening guidelines do not support regular screening of women at younger ages than 50 years, thereby largely excluding potentially high-risk women from screening in younger age groups. The relationship between the benefits and potential harms of screening in the age group under 50 years has been controversial and screening is not recommended for all women under age 50. The application of personalized risks is necessary to identify those women who could benefit from the earlier start of the screening. Also, for high-risk women over age 50 current screening with 2 intervals is not optimal and may miss interval cases.

A systematic review has found that "current trial data are too limited to directly inform the question of what the best screening strategy is for women or how clinicians can best tailor that strategy to the individual" (9). Age is an imperfect marker for risk, given that genetic susceptibility, lifestyle factors, and reproductive history can impact a woman's chance of developing BC. Risk-based screening, in which individualized risk assessment is used to inform screening practices, has been proposed as an alternative to age-based screening (10, 11). The UK dual cohort study showed that by introducing DNA data and mammographic density (MD, screening protocols could be stratified. Those at an estimated greater risk of high-stage cancers could be selected for enhanced screening (12).

It becomes evident that the current „one-size-fits-all" model for BC screening does not consider individual risks of women and is therefore not optimal. In the before-mentioned context, BC screening should be tailored to individual risks; therefore, there is a need for developing and standardizing tools for personalized risk estimation (13).

3.4. Tools for BC risk prediction

Breast density (BD)

BD is a well-established risk factor for BC, women with high density have an increased risk of developing BC (14, 15). It is usually quantified as mammographic density (MD), the proportion of fibroglandular and connective tissue relative to fatty tissue in the mammogram. MD is also a risk for masking the disease. Despite the long-time availability as a BC risk factor, BC screening is not so far personalized according to individual BD of women.

MD is routinely registered in the Norwegian Breast Cancer Registry database for all women diagnosed with BC and for all women recalled from screening for further assessment. MD is not registered for women who are negatively screened. For women who undergo clinical mammography, registration of MD is not obligate, but in VestreViken MD is usually classified and described in the report. We use the BI-RADS 5th edition categories a-d:

Category	Description

BI-RADS a	Almost entirely fatty
BI-RADS b	Scattered areas of fibroglandular density
BI-RADS c	Heterogeneously dense, which may obscure small masses
BI-RADS d	Extremely dense, which lowers the sensitivity of mammography

As of today, no formal recommendation exists regarding follow-up of women with dense breasts in clinical mammography or screening. However, in clinical mammography, the need for supplementary imaging is evaluated on an individual basis by the radiologists, taking clinical information and risk factors into account.

Genetic predisposition

Approximately 15 to 20 percent of BC is familial, meaning the disease occurs more often in family members than can be expected in the general population even though a particular gene mutation has not been identified in the family. With familial BC, the specific cause of BC is unknown but is likely due to combinations of risk factors, including genetics, lifestyle, and environment that increase risk in the family. Family history, as modelled in BC combined risk models, modulates the actual cancer risks, and may be used for further management decisions (16).

On the other side, around 30% of the total BC risk has been shown as hereditary (17). Genetic factors include monogenic pathogenic variants (MPV) in high and moderate-risk cancer predisposition genes (*BRCA1*, *BRCA2*, etc.), having effects large enough to warrant monogenic testing (18-20). However, only a fraction (5-10%) of BC cases are caused by these rare MPVs (21). A considerable part of BC variation is explained by variants outside these high-risk genes in the form of BC-associated common single-nucleotide polymorphisms (SNPs) (22, 23). It has become possible to aggregate information across many common SNPs in the form of polygenic risk scores (PRS) to predict disease risk and to develop applications with potential clinical utility. A PRS is the combined effect of individual BC susceptibility SNPs identified by genome-wide association studies. Although individual associated SNPs may confer only modest disease risk, the combined effect of all known associated SNPs on risk can be substantial. BC PRSs identify differences in genetic risks and provide a straightforward basis for designing personalized screening programs by accounting for individual genetic susceptibility (24). Currently, PRSs have not yet been implemented in routine BC screening, but simulations have suggested that risk profile informed behavioral adjustments could provide cost savings to screening and guide risk-based follow-up actions (25, 26). High-risk estimation could be also the indication for the use of hormonal chemoprevention (27).

There is so far no agreed clinical model for the implementation of PRS in BC personalized screening (13).

High and Intermediate Penetrance Susceptibility Genes – Monogenic Pathogenic Variants (MPVs)

Genetic factors include pathogenic mutations in high and moderate-risk cancer predisposition genes (*BRCA1*, *BRCA2*, *ATM, CHEK2, TP53, PTEN, STK11, CDH1, PALB2, NBN, NF1,* and *BARD1*), having effects large enough to warrant monogenic testing (18-20). Pathogenic germline mutations in the BRCA1 and BRCA2 genes dramatically increase the relative risk of BC, particularly among premenopausal women. For example, the cumulative lifetime risks of female BC may be as high as 46-87 % for BRCA1 and 38-84 % for BRCA2 (28). A recent prospective cohort study showed that the cumulative risk of developing BC by the age of 80 years was 72% for *BRCA1* mutation carriers and 69% for *BRCA2* mutation carriers, respectively (16). For ovarian cancer, the cumulative risks by age 80 years were 44% for *BRCA1* carriers and 17% for *BRCA2* carriers. BC incidence for carriers increased rapidly with age in early adulthood and then remained relatively constant throughout the lifetime. The age at which this “plateau” was reached

was 31 to 40 years for *BRCA1* carriers and 5 to 10 years later for *BRCA2* carriers. The incidence during the plateau was similar for both groups of mutation carriers (16).

Pathogenic variants in other genes have been associated with BC risk including TP53, PTEN, CDH1, STK11, and PALB2 being associated with high risk (>40% lifetime), and CHEK2 and ATM moderate risk (20–30% risk).

Currently, the clinical genetic testing for BC susceptibility is focused on detecting pathogenic mutations within known high- and moderate-risk genes, based on the family background of cancers, especially cancers at a young age. However, only a fraction (4-10%) of breast cancer cases are caused by these rare genetic variants (21), and the majority of women who develop breast cancer do not have a pathogenic variant in an actionable gene.

Current practices of BC MPV testing in Norway
Current criteria for MPV testing are characterized here:

https://www.helsebiblioteket.no/retningslinjer/brystkreft/forord

Criteria for genetic testing of BC patients:

- Women diagnosed with BC 60 years or younger.
- Male BC any age.
- Women diagnosed with ovarian cancer at any age.
- Women diagnosed with BC older than 60 years with a family history of cancer as follows:

1) One first-degree relative (or second-degree through a man) with BC, and the mean age of onset is 55 years or younger.

2) Two first-degree relatives (or second-degree through a man) with BC at any age.

3) One first-degree relative (or second-degree through a man) with ovarian cancer.

4) One first-degree relative with prostate cancer 55 years or younger.

A healthy woman can be offered genetic testing if she has a first-degree relative who fulfills any of the criteria above.

Follow up of women with an increased familial risk for BC:

Women with a mutation in the high-penetrance genes are offered risk-reducing surgery with prophylactic mastectomy. High-risk women who have not removed their breasts are offered annual screening with mammography and breast MRI from the age of 25 until the age of 70 for women with BI-RADS density category c or d, and 60 for women with a density a or b. Above this age (60/70), the women are offered annual mammography as long as considered appropriate according to general health status.

Women considered to have an increased familial risk for BC without mutations in the high-penetrance genes are recommended annual mammography from 40-60 years. From the age of 50, they are screened every second year in the ordinary screening program and referred for clinical mammography the years

in-between. From the age of 60, they are recommended to participate in the ordinary biennial screening program. No special follow-up is recommended after the age of 70.

Polygenic risk score

A considerable part of BC variation is explained by variants outside these high-risk genes in the form of BC-associated common single-nucleotide polymorphisms (SNPs) (22).

A polygenic risk score (PRS) is the combined effect of individual breast cancer susceptibility variants identified by genome-wide association studies. Although individual associated SNPs may confer only modest disease risk, the combined effect of all known associated SNPs on risk can be substantial. Under the assumption of a simple multiplicative model, the collection of known susceptibility SNPs for any given trait or disease can be combined into a risk profile that can be used for risk prediction.

Several studies have combined SNPs with genome-wide significance into a summary estimate of BC risk using a PRS approach (23, 29, 30). Initial attempts by Mavaddat et al. (23) and Sieh et al. (31) demonstrated a strong effect of the score in predicting future BC cases. Other major efforts such as those by Mavaddat et al., Khera et al., and Hughes et al. additionally elucidated the use of PRS as a BC predictor (29, 32, 33).

BC PRS tests are not yet widely implemented in healthcare.

Polygenic Risk Score (PRS) test AnteBC

AnteBC is a genetic test that assesses a woman's personalized risk of developing breast cancer (BC) using a polygenic risk score (PRS).

Health technology company Antegenes (License number L04685) has developed AnteBC as a laboratory test. AnteBC is registered as a medical device (IVD) in Estonian Medical Devices Database (EMDDB code: 14726).

For the PRS calculation, AnteBC uses the patient’s DNA data from genotyping. Genotyping is performed using the AnteBC test with a non-invasive saliva-based method to collect and isolate a patient’s DNA. The standard procedure includes the use of Illumina Global Screening Array-24 (GSA) v3.0 chip and Illumina iSCAN sequencer for genotyping. This workflow genotypes the ~762 000 markers on the GSA chip by following the Illumina Infinium HTS (high-throughput screening) protocol (Illumina Inc, http://www.illumina.com; Document # 15045738 v04).

The test can also use information from other microarrays and sequencing approaches that output DNA data broadly covering the human genome.

Antegenes performs the risk assessment based on imputed genotype data. Quality-controlled markers resulting from genotyping are imputed using a 1000G panel with reference to the human genome GRCh37.

Aim of the AnteBC test

The purpose of the AnteBC test is to reduce the risk of premature mortality due to BC through stratified precision BC screening and other preventive measures. AnteBC is a tool for individual BC risk estimation, which is the basis for more precise and efficient prevention. The aim is to evaluate the genetic

predisposition for BC with PRS technology (AnteBC test) and detect women with moderately or highly raised BC risk for stratified screening.

AnteBC clinical indications

Target population: AnteBC test is recommended for women between the ages of 30 and 75 of primarily European ancestry, but the test has also corrections for other ethnicities based on analyses of risk performance with UK Biobank ethnic groups' data.

BC risk prediction models

The classical BC risk prediction models provide risk estimates by either non-genetic risk factors (demographics, reproductive history, menopausal status, family history, breast biopsies, and mammographic density) (34, 35) or by penetrance data from high-risk families with BRCA1 and BRCA2 mutations (36, 37).

QCANCER (http://www.qcancer.org/female/index.php) requires age, smoking, alcohol consumption, familial history of various cancers, other diseases (T2D, COPD), etc. This and other non-genetic models have a limited utility on large scale, as up-to-date information about risk factors is not available. QCancer® has been developed for the UK population and is intended for use in the UK.

The **Claus model** includes first- and second-degree relatives with breast and/or ovarian cancer, and incorporates the age at diagnosis (38). Among the limitations of the Claus model are that it applies only to women with a family history of BC and does not consider nonhereditary risk factors. In addition, the Claus model can calculate risk for only up to two relatives with BC.

The **BRCAT or Gail model** (https://www.cancer.gov/bcrisktool/) is primarily focused on personal risk factors for BC and estimates the patient's five-year and lifetime risk of BC. The five-year risk can be used for chemoprevention recommendations if the risk is greater than 1.67 percent. The main limitation of the Gail model is that it includes only first-degree relatives, which underestimates the risk in women with BC history in their paternal lineage. The Gail model also does not consider the age of onset of BC. These risk calculations have been validated for women in the U.S. who are screened regularly for BC.

The **BRCAPro** (http://bcb.dfci.harvard.edu/bayesmendel/brcapro.php) (39) considers a patient's age, the age of first- and second-degree relatives and their breast and ovarian cancer status, along with *BRCA1* and *BRCA2* mutation frequencies and cancer penetrance. The patient's ethnicity and age at which she has had a salpingo-oophorectomy (the removal of the ovaries and Fallopian tubes) are also components of the BRCAPRO risk calculation. This model does not consider nonhereditary risk factors.

The Breast Cancer Surveillance Consortium (BCSC) model (https://tools.bcsc-scc.org/BC5yearRisk/intro.htm) was developed and validated in 1.1 million women undergoing mammography across the United States, among whom 18,000 were diagnosed with invasive breast cancer (40).

The **BOADICEA** (the Breast and Ovarian Analysis of Disease Incidence and Carrier Estimation Algorithm) (http://ccge.medschl.cam.ac.uk/boadicea/) (41, 42) is a comprehensive model that can be used to calculate the future risks of developing breast or ovarian cancer using the information on family history, lifestyle/hormonal risk factors, rare pathogenic variants in moderate and high-risk breast/ovarian cancer

susceptibility genes, common breast/ovarian cancer genetic susceptibility variants (PRS) and mammographic density. It can also be used to calculate the likelihood of carrying mutations in the moderate to high-risk genes BRCA1, BRCA2, PALB2, ATM, CHEK2, BARD1, RAD51C, and RAD51D (43).

The **CanRisk Web Tool** incorporates the new version of BOADICEA v8, the Breast and Ovarian Analysis of Disease Incidence and Carrier Estimation Algorithm. https://www.canrisk.org

The **Tyrer-Cuzick model** (the International BC Intervention Study [IBIS]) (http://www.ems-trials.org/riskevaluator/) (44) considers the patient's age; BMI; age at first menstrual period; age at first live birth; age at menopause; hormone replacement therapy use; breast biopsies; atypical ductal hyperplasia; lobular carcinoma in situ; and family history of breast and ovarian cancer in first- and second-degree relatives. This model also estimates the risk for the presence of a non-*BRCA1*/*BRCA2* BC susceptibility gene mutation. The program assumes that there is a gene predisposing to BC in addition to the *BRCA1/2* genes. The risk from other classical factors including age at a first child and benign disease is combined with familial risk.

For risk-based screening, combined risk models must be easily accessible and user-friendly, but as medical devices for medical decisions according to European Union regulations, they should be compliant with relevant healthcare regulations. The practical routine application of such compounded models has been complicated due to the non-availability and quality of data about individual risk factors. In practical settings, the data collection difficulties need to be weighed with expected gains.

3.5. BC Risk Categories

The first step in determining a risk category is to assess for major risk factors to identify women at average risk, which is the category for most women and to identify the smaller number of women at moderate or high risk of developing BC. The risk categories are delineated according to the lifetime risk of being diagnosed with BC (not according to the risk of dying due to BC). There is no standardization or consensus about the exact percentages of lifetime risk of developing BC within each risk category.

Major factors used to determine a risk category, based on a patient’s history, are:

- Personal history of ovarian, peritoneal (including tubal), or BC.
- Family history of breast, ovarian, or peritoneal cancer.
- Genetic predisposition (if the patient’s *BRCA* or another genetic marker status is known).

Women who have none of these risk factors are usually considered at average risk. Most women are in this average-risk category, with an average lifetime risk of being diagnosed with BC estimated at 12.4 percent in the USA population.

The study by Maas et al. estimated the average absolute risk for a 30-year-old white woman in the United States to develop invasive breast cancer by the age of 80 years at 11.3% (45). A model that includes all risk factors provided a range of average absolute risk from 4.4% to 23.5% for women in the bottom and top deciles of the risk distribution, respectively. For women who were at the lowest and highest deciles of nonmodifiable risks, the 5th and 95th percentile range of the risk distribution associated with 4 modifiable factors were 2.9% to 5.0% and 15.5% to 25.0%, respectively. The percentage of the population could be divided to be of moderate risk (twofold to threefold risk

compared with the population average) and high risk (above threefold risk compared with the population average).

In the review by D. Easton et al. risk levels are defined as: moderate risk as a risk of breast cancer, defined in terms of disease incidence, that is two to four times as high as that in the general population, and high risk as an incidence that is more than four times as high (46).

In the WISDOM study for women aged 40 to 49 years, screening is recommended when their five-year risk equals or exceeds that of the average woman aged 50 years (47). They based thresholds on five-year risk given that screening and prevention are most impactful in those at immediate risk of cancer, and five-year risk thresholds are standardly used to guide chemoprevention. The 5-year risk estimate in the WISDOM study for women aged 50 was 1.3%.

UK National Institute for Health and Care Excellence (NICE) guidelines for the management of familial BC define BC risk categories using the following thresholds (48), Table 8 below:

Table: BC risk categories by NICE guidelines

	BC risk category		
	Near population risk	Moderate risk	High risk
Lifetime risk from age 20	Less than 17%	17% or greater but less than 30%	30% or greater
Risk between ages 40 and 50	Less than 3 %	3-8%	Greater than 8%

Also, the following approach has been used: generally, they are as follows: average (less than 15 percent), moderate (approximately 15 to 20 percent), or high (greater than 20 percent) lifetime risk.

PRS methodology uses the normal statistical distribution of risks, therefore PRS risks distribution has no clear different thresholds. For the clinical implementation of the PRS test, Antegenes has developed clinical recommendations using based mostly on two aspects – the average BC risk level currently accepted for routine BC public screening and the analogy with clinical recommendations for moderate-risk MPV carriers(49)(50). For risk-based recommendations based on the PRS, it is possible to divide the patient's relative risk of developing BC into different levels compared to the average in the given age, while accurately assessing the risk of a particular percentile. In Europe, mammography screening in the age group 50-69 years at two-year intervals is currently a recognized standard practice, which reduces BC mortality. Consequently, the “zero point” of the risk level at the beginning of the screening is the average risk level of 50-year-old women. Detecting younger women with similar or higher risk levels already from age 30-to 35 allows implementing for them similar mortality reduction measures, avoiding same time screening measures for women with lower risk. In the case of different standard-screening groups (for example, screening from age 40), it is possible to apply accordingly modified recommendations. As PRS can predict similar BC risk levels to moderate risk MPVs (in genes *ATM, CHEK2,* and others), we used for our recommendations analogy with these for mammography intervals and magnet resonance imaging use(49)(50).

3.6. Cost-effectiveness data of personalized risk-based screening

Simulation models have suggested that tailoring screening based on individual risk factor profiles may be more cost-effective than uniform screening (10) and using genetic risk thresholds rather than age may more efficiently identify candidates for screening (50). A systematic review with a focus on the cost-effectiveness of risk-adapted strategies concludes that e that BC screening and prevention are cost-effective in the European settings (51). All screening models predict gains in life expectancy, which has not yet been confirmed by trials with long follow-up periods. European models evaluating risk-adapted screening strategies are rare but suggest that risk-adapted screening is more effective and cost-effective than conventional screening (51).

3.7. Background Summary and Study Rationale

The current 'one-size-fits-all' BC screening approach does not consider individual risks of women and there is a need for more precise screening which considers individual risks of women.

BC screening is widely implemented in many healthcare systems to reduce BC mortality through earlier diagnosis. Also, in Norway screening is offered to women in age 50-69 with mammography after every 2 years. Screening with mammography reduces BC mortality risk 20-40% among women at age 50-69. But approximately 20% of BCs occur in women younger than 50 years. The relationship between benefits and potential harms for screening in the age group under 50 years has been controversial and, in most countries, as well as in Norway, screening is not offered in this risk group.

For a personalized approach, we need tools for risk stratification. Around 30% of the total BC risk has been shown as hereditary, therefore genetic predisposition tests serve as tools for risk estimations. These tools are already in clinical use in the form of polygenic risk score (PRS) tests and monogenic pathogenic variants' (MPV) tests, and these can serve as BC risk prediction instruments for personalized prevention. Also, BD in mammography is a tool for BC risk prediction and more precise recommendations for screening. We have developed a new model for a genetic risk-based BC screening program that enables personalized prevention based on individual risk levels.

The screening plan should depend on a woman's risk for BC and personal preferences. The use of genetic risk scores and mammographic BD identifies women that are at increased risk of developing the disease.

Risk assessment per se does not have inherent clinical utility; the subsequent adoption of a risk-based intervention based on the results of the assessment is what influences the health outcomes. The use of such a strategy depends on whether the risk-based intervention is appropriate, accessible, practicable, and acceptable. To combine individual genetic risk testing, BD information and subsequent healthcare services, it is necessary to create and implement a whole program of personalized genetics-based BC screening and prevention. It is necessary to evaluate: the benefit–harm trade-offs of such strategies and the acceptability and feasibility of implementing risk-stratified prevention and early detection programs.

The current project is aimed to investigate these aspects as a comprehensive program of personalized BC prevention and screening.

Key hypotheses:

- Genetics-based BC prevention program has a clinical utility and is cost-effective for women under age 40 or 50.

- Genetics-based BC prevention program is feasible and acceptable, and healthcare systems are ready to implement that.

Research questions also are:

- What is the clinical utility and effectiveness of personalized BC screening and prevention?
- What is the cost-effectiveness of genetics-based personalized precision prevention?
- Is genetics-based personalized precision prevention feasible in clinical practice?
- What are the acceptability, values, preferences, and social norms for personalized precision prevention?
- Is the accessibility of genetics-based personalized precision prevention equitable?
- How ready are the healthcare systems for the implementation of personalized precision prevention as a population-based approach?

4. OBJECTIVES AND OUTCOME MEASURES

The general aim is to perform a clinical cohort study that would test issues regarding the deployment of PRS test AnteBC and MPV-based risk testing in the Norwegian clinical setting. This clinical pilot would simultaneously evaluate logistical and ethical considerations (patient risks, feedback, operational procedures), test the flow of information, and would also create prospective performance data for PRS testing. In addition, the project will explore the relationship between PRS and mammographic density (MD).

The target group is healthy women at age 40-49, which is the age group before the current official screening. For genetic risk prediction, the project uses PRS and, if indicated, MPV tests as healthcare services with the following diagnostic screening and prevention activities.

Primary aim:

To evaluate the impact of implementing population-based genetics testing strategy for BC precision prevention using the PRS and MPV testing.

Measurable primary outcome:

Elevated-risk BC PRS and MPVs prevalence for precision screening - the proportion of women with elevated risk for personalized screening.

The proportion of women detected with higher genetic or breast density risk for BC screening according to:

- PRS risk.
- Monogenic risk.
- Based on high BD on mammography.

Secondary study aims:

To evaluate the feasibility, potential clinical utility, and cost-effectiveness of a population-based genetic testing strategy for BC precision prevention.

Secondary outcomes:

- Clinical utility and effectiveness of the genetics-based personalized precision prevention.
- Cost-effectiveness of the genetics-based personalized precision prevention.
- Feasibility of the genetics-based personalized precision prevention.
- Acceptability, values, preferences, and social norms for personalized precision prevention.
- Equitable accessibility of genetics-based personalized precision prevention.
- Health-care system readiness for the implementation of personalized precision prevention.
- Results of high and moderate risk genetic testing (BRCA1, BRCA2, TP53, STK11, PTEN, CDH1, ATM, PALB2, CHEK2, NBN, NF1).
- Results of SNPs testing and SNP PRS analyses.
- PRS risk versus breast density.
- Individuals' distribution into risk groups.
- Individuals' participation rate from additional counselling; analysis of patients' compliance.
- Exact analysis and description of medical activities after counselling.
- The number of family members invited to additional counselling and their participation rate.
- Costs analysis of all activities.

Effectiveness and clinical utility of the genetics-based personalized precision prevention.

Clinical utility refers to the usefulness, benefits, and harms of an intervention. Clinical utility is a multidimensional construct covering effectiveness and cost-effectiveness, as well as the psychosocial, ethical, and legal implications of an intervention. Risk assessment per se does not have inherent clinical utility; the subsequent adoption of a risk-based intervention based on the results of the assessment is what influences the health outcomes. The use of such a strategy depends on whether the risk-based intervention is appropriate, accessible, practicable and acceptable.

Cost-effectiveness of the genetics-based personalized precision prevention.

We will use modelling of cost-effectiveness with input data from the clinical pilot project, epidemiological data, relevant healthcare services and their cost data.

Feasibility of the genetics-based personalized precision prevention

Feasibility will be investigated by conducting the clinical pilot project describing the process, organization, and results of the applied model of personalized precision prevention.

Acceptability, values, preferences, and social norms for personalized precision prevention

Acceptability and preferences will be investigated using relevant questionnaires for:

- Women included in the clinical pilot project.
- Medical personnel involved in processes of personalized prevention.

Equitable accessibility of the genetics-based personalized precision prevention

Equitable accessibility will be investigated by conducting the clinical pilot project and describing the process, organization, and results of the applied model of personalized precision prevention.

Healthcare system readiness for the implementation of personalized precision prevention

Healthcare system readiness will be investigated by conducting the clinical pilot project and describing the process, organization, and results of the applied model of personalized precision prevention.

5. STUDY DESIGN

General population-based cohort study combined with modelling studies.

The target number of study participants: 80.

5.1. Study Participants

Inclusion Criteria:

- A study group of women at age 40-49 (women currently not invited into regular BC screening), who are referred to clinical mammography and no sign of malignancy after mammographic assessment.

Exclusion Criteria:

- Women with already diagnosed malignancies or hereditary cancer syndromes.
- Signs of malignancy after mammographic assessment.
- Women already tested for MPVs and PRSs.
- Ashkenazy Jewish ethnicity.

Research methodology

We will use a pilot feasibility cohort study approach, collecting data from the study and adding modelling clinical efficacy and cost-effectiveness analyses.

The pilot feasibility study implements the program of the genetics-based personalized BC prevention and screening for healthy women.

5.2. STUDY PROCEDURES AND RECRUITMENT

Women referred to clinical mammography and no sign of malignancy after assessment, will be asked to participate in the study. These women are referred to mammography due to clinical symptoms, like breast lumps, pain, and nipple discharge. Women referred due to symptoms may have an increased risk for BC, compared to women without symptoms. Further, women with dense breast tissue have a higher risk of BC than women with less dense breasts.

Participation in the study is not solely limited to women referred to clinical mammography in Vestre Viken. However, we will not actively recruit women outside the Vestre Viken hospital area, but may include these women after a personal request from the women.

STEP 1. Information

Information about the genetic-based BC precision prevention possibilities is provided to the women at the breast centre.

The women will be asked for study participation and asked for informed consent for personalized screening and prevention.

STEP 2. Genetic testing / Risk evaluation

A recommendation on the level of the genetic test is given, variants:

- All women: PRS testing. Genotyping is performed using the AnteBC test with a non-invasive saliva-based method to collect and isolate a patient's DNA. Antegenes performs the risk assessment based on imputed genotype data.
- In addition: questionnaire about genetic and cancer family history.
- Women that fulfill standard criteria according to national guidelines will be submitted to genetic counselling and testing of monogenic germline pathogenic variants (MPV), see chapter 3.2 for criteria.

After the patient's **informed consent**, an AnteBC test is performed and, if indicated, a patient is directed also for **MPV testing**.

STEP 3. Recommendations based on results of genetic testing/risk evaluation

Recommendations based on PRS test:

Based on the PRS, it is possible to divide the patient's relative risk of developing BC into different levels compared to the average in the given age, while accurately assessing the risk of a particular percentile.

Different Levels of Disease Risk:

- Lower or at the same level.
- Slightly elevated (up to two times).
- Moderately elevated (two to three times).
- Elevated more than three times.

Applying the logic of the model:

In Norway, mammography screening in the age group 50-69 years at two-year intervals is currently a recognized standard practice. Consequently, the "zero point" of the risk level at the beginning of the screening is the average risk level of 50-year-old women. We can conclude that currently in Norway BC screening mammography is an accepted and publicly funded early BC detection measure for women in the age group 50-69, where the average BC 10-year risk at age 50 is 2.1% using NORDCAN data (https://www-dep.iarc.fr/NORDCAN/english/frame.asp) and iCARE absolute risk calculation (7).

Patient's individual and patient's population average 10-year BC risks are reported in the AnteBC test report.

When assessing individual risk levels, the above risk groups can be advised based on current scientific knowledge:

Variant 1. If the risk is below average or at a medium level:

- Participate in a standard mammography screening from the age of 50.

Variant 2. If the risk is slightly increased - up to two times (moderate increase in risk depending on age):

- Implement mammography screening at **two-year intervals** from the age at which the risk of the average 50-year-old woman is reached (depending on the age at which the 10-year risk reaches 2.1 %), i.e. screening is recommended for those under 50 years of age due to an increased risk.

Variant 3. If the risk is increased two to three times (moderate increase in risk compared to the same age average):

- Implement mammography screening at **two-year intervals** from the age at which the risk of the average 50-year-old woman is reached (depending on the age at which the 10-year risk reaches 2.1 %.

and/or

- Implement mammography screening at one-year intervals from the age at which the average 50-year-old woman reaches twice the risk level (depending on the age at which the 10-year risk exceeds twice the corresponding age level or for women under 50 from the risk level of 2 x 2.1 %, which is twice the risk level for women aged 50);

Variant 4. If the risk has increased more than three times the average (high-risk increase):

- Implement mammography screening at **two-year intervals** from the age at which the risk of the average 50-year-old woman is reached (depending on the age at which the 10-year risk reaches 2.1 %)

and/or

- Implement mammography screening at one-year intervals from the age at which the average 50-year-old woman reaches twice the risk level (depending on the age at which the 10-year risk exceeds twice the corresponding age level or for women under 50 from the risk level of 2 x 2.1 %, which is twice the risk level for women aged 50);

Recommendations based on MPV test:

No MPVs detected: approach according to **the PRS test recommendations**.

MPV detected: additional genetic counselling and approach according to **the national guidelines for MPV management (see Chapter 3.2.)**:

- Patient counselling by a medical geneticist, family mapping, and cascade testing.
- The patient is referred to a gynecologist/breast cancer specialist according to national guidelines.
- Treatment of increased risk at the health care provider level according to national guidelines: personalized prevention is implemented.

Recommendations based on mammographic BD information

In current clinical practice, no formal recommendation exists regarding follow-up of women with dense breasts in clinical mammography or screening. However, in clinical mammography, the need for supplementary imaging is evaluated on an individual basis by the radiologists, taking clinical information and risk factors into account.

In this study, women with all breast density categories will receive recommendations based on the PRS score (or national guidelines for MPV management if MPV is detected).

STEP 4. Implementation of personalized screening and prevention (risk reduction)

Next steps after test results

Personalized Prevention Measures Based on the AnteBC test

Based on the PRS, it is possible to divide the patient's relative risk of developing BC into different levels compared to the average in the given age, while accurately assessing the risk of a particular percentile. The women will be recommended annual or biennial mammography examination according to risk level and Variant 1-4 listed in Step 3.

The population-based screening program has a central organization with a standardized regime for an invitation, screen-reading, and registration of screening results. Modification of screening logistics as entry age or screening frequency is not possible within the screening program as such. Thus, for women recommended mammographic screening starting at an earlier age (<50) and/or increased frequency (annual screening), these "extra" examinations will be offered as a clinical mammography examination outside the screening program.

The women will be followed for 10 years.

5.3. BLODD SAMPLES/BIOBANK

The women participating in the pilot for PERSONALIZED RISK-BASED BREAST CANCER SCREENING will also be asked to give a wide consent to general biobank for breast cancer in Vestre Viken (REK number 108572). The biobank includes also women that are assessed for breast cancer and precursors for breast cancer. Women that do not have breast cancers are included as controls. The blood samples will be stored for future research. The women can participate in the study without giving consent to general biobank breast cancer. The women will be asked to consent to registry and biobank through a separate box. The consent for general biobank and registry will be given to the women for information, but the women will only sign the pilot study.

5.4. ASSESSMENTS

Baseline Assessments

- Age
- Date of mammography
- Result of mammography: normal / needs additional specification / tumour
- MD
- Biopsy
- Confirmed invasive cancer
- Confirmed in situ carcinoma
- PRS level

In case of PRS only:

- Age
- Personal medical history

- Family cancer history
- Current medications (hormonal agents)
- Mammography: normal / needs additional specification / tumour
- MD
- Short- and long-term psychosocial aspects related to genetic feedback (questionnaires after the return of data and after 6 months)

In case of monogenic pathogenic variants:

- Age
- Mutated gene
- Type of mutation
- Personal medical history
- Family cancer history
- Description of cancer risk levels
- Short- and long-term psychosocial aspects related to genetic feedback (questionnaires after the return of data and after 6 months)
- Current medications (hormonal agents)
- Mammography
- MD
- MRT

Variables Collected

- Name
- Personal ID
- Date of birth
- Age
- Phone
- E-mail
- Postal address
- Preferred contact channel
- Family history for monogenic pathogenic variants (MPV)
- Family history of cancers
- Polygenic risk score (PRS) test results
- MPV test results

- Date of mammography
- Result of mammography: normal / needs additional specification / tumor
- Mammographic BD
- Date of biopsy, if performed
- Results of biopsy: Benign / Confirmed invasive cancer / Confirmed in situ carcinoma
- Occurrence and date of medical counselling

  In the case of MVPs:
  - Date of clinical geneticist consultation
  - Date of oncologists' consultation

  - Date of mammography
  - Result of mammography: normal / needs additional specification / tumor
  - Mammographic BD
  - Date and results of MRT
  - Date and results of abdominal and gynecological ultrasound
  - Date and results of CA125; HE4, if performed.

- Information about possible hormonal chemoprevention (when started, what drug)
- Information about possible risk-reducing surgeries (when and what)
- Information about possible detected malignancies: Date, type, stage, TNM, morphology
- Short- and long-term psychosocial aspects related to genetic feedback (questionnaires after the return of data and after 6-9 months).

5.5. Discontinuation/Withdrawal of Participants from Study

Each participant has the right to withdraw from the study at any time.
The reason for withdrawal will be recorded in the eCRF.

5.6. Definition of End of Study

Activities with patients for study data collection are planned to finish after 10 years of follow-up.

6. STATISTICAL METHODS

6.1. Description of Statistical Methods

Data will be mainly analysed by descriptive statistics.

6.2. Analysis of Outcome Measures

Planned data for outcome analyses:

- Study group characteristics: age, distribution of age groups, and other clinical characteristics.

Additional outcomes:

- Results of SNPs testing and SNP PRS analyses.
- Individuals’ distribution into risk groups.
- Individuals’ participation rate from additional counselling; analysis of patients’ compliance.
- Exact analysis and description of medical activities after counselling.
- Association of PRS score and breast density

Current Terms of Reference for the Study, and study time frame, do not allow for conducting a randomized long-term study, which could be capable to analyse real changes in incidence and mortality of BC.

Cost-effectiveness analyses

During the final stage (last 6 months) of the pilot study, the cost-effectiveness analysis will be conducted.

The cost-effectiveness analysis will aim to estimate the difference in health outcomes and the difference in costs of implementing the personalized medicine interventions as compared to standard care.

Methodology of the cost-effectiveness study

Whereas the whole pilot study aims to develop models for the personalized medicine interventions, it is not possible to predict which efficacy and effectiveness measures will be best suited to estimate the health outcomes of the program.

The planned personalized medicine interventions are preventive, and the pilot project will last less than 2.5 years, thus the most robust health outcome of mortality cannot be achieved during the study. Therefore, additional health outcomes, e.g. disease-free time to diagnosis and/or hospital care, stage of cancer diagnosed will be used to estimate the effect of preventive measures to increase the disease-free life years, postpone the negative health events, and decrease the need to use health care resources will be collected. This data will be used to model the number of events occurring in specified cohorts of persons at risk – a cohort receiving personalized preventive interventions will be compared to that on standard care.

To estimate the long-term effectiveness and costs of the new interventions, economic modeling will be used. The Markov cohort models of the natural history of BC will be constructed to assess the cost-effectiveness of preventive interventions.

The recently published cost-effectiveness studies modelling BC will be used to construct the natural history of the disease. Annual age-specific probabilities of dying from this disease and the health-related quality of life decrement during the illness will be calculated for the Swedish, Estonian and Portuguese cohorts using published cost-effectiveness studies.

The analysis results will be presented as incremental costs and quality-adjusted life-years (QALYs) gained with each intervention compared to no personalized medicine and as incremental cost-effectiveness ratios (ICERs). Both costs and utilities will be discounted at an annual rate of 5%. The sensitivity analysis will be performed by varying one or more similar parameter(s) at a time while holding other parameters at their base case value.

The data used for cost calculations will include

- Direct costs of the use of health care services (out-patient care, hospital care, and pharmaceuticals), based on the data from healthcare systems.

- Costs for developing and maintaining IT solutions for collecting and using health information for personalized medicine purposes.

- Costs for collection and analysis of additional samples arising from the new clinical approaches of the two preventive interventions.

- Costs for disseminating the results of the pilot project to the scale of the whole population: training of health care staff and additional workload estimates related to full implementation of the personalized medicine approach (e.g. motivational interviewing).

Most of this cost data will be collected in parallel to the implementation of the pilot project.

6.3. Access to Data

Direct access will be granted to authorized representatives from the Sponsor and host institution for monitoring and/or auditing of the study to ensure compliance with regulations.

7. QUALITY ASSURANCE PROCEDURES

We plan data quality monitoring inside the study group, no data monitoring is planned from the third parties.

8. ETHICAL AND REGULATORY CONSIDERATIONS

8.1. Declaration of Helsinki

The Investigator will ensure that this study is conducted following the principles of the Declaration of Helsinki.

8.2. Guidelines for Good Clinical Practice

The Investigator will ensure that this study is conducted following relevant regulations and with Good Clinical Practice.

8.3. Approvals

The protocol, informed consent form, participant information sheet and any proposed advertising material will be submitted to an appropriate Research Ethics Committee (REC for written approval.

The Investigator will submit and, where necessary, obtain approval from the above parties for all substantial amendments to the original approved documents.

8.4. Reporting

The CI shall submit once a year throughout the study or on request, an Annual Progress report to the REC Committee and Sponsor. In addition, an End of Study notification and the final report will be submitted to the same parties.

8.5. Participant Confidentiality

The study staff will ensure that the participants' anonymity is maintained. The participants will be identified only by a participant ID number on all study documents and any electronic database, except the eCRF, where participant initials may be added. All documents will be stored securely and only accessible by study staff and authorized personnel. The study will comply with the Data Protection Act, which requires data to be anonymized as soon as it is practical to do so.

8.6. Expenses and Benefits

There are no planned payments for participants.

9. FINANCE AND INSURANCE

9.1. Funding

The project is funded by the Norway Grants "Green ICT" program and sponsored by the AnteNOR Consortium.

9.2. Insurance

No insurance is planned in the current project.

10. PUBLICATION POLICY

The Investigators will be involved in reviewing drafts of the manuscripts, abstracts, press releases, and any other publications arising from the study. Authorship will be determined following the ICMJE guidelines and other contributors will be acknowledged.

11. APPENDIX A: AMENDMENT HISTORY

Amendment No.	Protocol Version No.	Date issued	Author(s) of changes	Details of Changes made

12. REFERENCES

1. Sung H, Ferlay J, Siegel RL, Laversanne M, Soerjomataram I, Jemal A, et al. Global Cancer Statistics 2020: GLOBOCAN Estimates of Incidence and Mortality Worldwide for 36 Cancers in 185 Countries. CA Cancer J Clin. 2021;71(3):209-49.
2. Ferlay J EM, Lam F, Colombet M, Mery L, Piñeros M, Znaor A, Soerjomataram I, Bray F. Global Cancer Observatory: Cancer Today. Lyon, France: International Agency for Research on Cancer. Available from: https://gco.iarc.fr/today, accessed [28 May 2022] 2020 [
3. Myers ER, Moorman P, Gierisch JM, Havrilesky LJ, Grimm LJ, Ghate S, et al. Benefits and Harms of Breast Cancer Screening. Jama. 2015.
4. Tabár L, Dean PB, Chen TH-H, Yen AM-F, Chen SL-S, Fann JC-Y, et al. The incidence of fatal breast cancer measures the increased effectiveness of therapy in women participating in mammography screening. Cancer. 2018.
5. Marmot MG, Altman DG, Cameron DA, Dewar JA, Thompson SG, Wilcox M. The benefits and harms of breast cancer screening: an independent review. Br J Cancer. 2013;108(11):2205-40.
6. Sebuodegard S, Botteri E, Hofvind S. Breast Cancer Mortality After Implementation of Organized Population-Based Breast Cancer Screening in Norway. J Natl Cancer Inst. 2020;112(8):839-46.
7. Choudhury PP, Maas P, Wilcox A, Wheeler W, Brook M, Check D, et al. iCARE: R package to build, validate and apply absolute risk models. bioRxiv. 2018:079954.
8. Loberg M, Lousdal ML, Bretthauer M, Kalager M. Benefits and harms of mammography screening. Breast Cancer Res. 2015;17:63.
9. Siu AL, Force USPST. Screening for Breast Cancer: U.S. Preventive Services Task Force Recommendation Statement. Ann Intern Med. 2016;164(4):279-96.
10. Schousboe JT, Kerlikowske K, Loh A, Cummings SR. Personalizing mammography by breast density and other risk factors for breast cancer: analysis of health benefits and cost-effectiveness. Ann Intern Med. 2011;155(1):10-20.
11. Shieh Y, Eklund M, Sawaya GF, Black WC, Kramer BS, Esserman LJ. Population-based screening for cancer: hope and hype. Nature reviews Clinical oncology. 2016;13(9):550-65.
12. Evans G, Asteley S, Stavrinos P. Improvement in risk prediction, early detection

and prevention of breast cancer in the NHS Breast

Screening Programme and family history clinics: a dual cohort study 2016 [
13. Pashayan N, Antoniou AC, Ivanus U, Esserman LJ, Easton DF, French D, et al. Personalized early detection and prevention of breast cancer: ENVISION consensus statement. Nature reviews Clinical oncology. 2020;17(11):687-705.
14. McCormack VA, dos Santos Silva I. Breast density and parenchymal patterns as markers of breast cancer risk: a meta-analysis. Cancer Epidemiol Biomarkers Prev. 2006;15(6):1159-69.
15. Eng A, Gallant Z, Shepherd J, McCormack V, Li J, Dowsett M, et al. Digital mammographic density and breast cancer risk: a case-control study of six alternative density assessment methods. Breast Cancer Res. 2014;16(5):439.
16. Kuchenbaecker KB, Hopper JL, Barnes DR, Phillips KA, Mooij TM, Roos-Blom MJ, et al. Risks of Breast, Ovarian, and Contralateral Breast Cancer for BRCA1 and BRCA2 Mutation Carriers. Jama. 2017;317(23):2402-16.
17. Mucci LA, Hjelmborg JB, Harris JR, Czene K, Havelick DJ, Scheike T, et al. Familial Risk and Heritability of Cancer Among Twins in Nordic Countries. Jama. 2016;315(1):68-76.
18. Frey JD, Salibian AA, Schnabel FR, Choi M, Karp NS. Non-BRCA1/2 Breast Cancer Susceptibility Genes: A New Frontier with Clinical Consequences for Plastic Surgeons. Plastic and Reconstructive Surgery – Global Open. 2017;5(11):e1564.

19. Wunderle M, Olmes G, Nabieva N, Haberle L, Jud SM, Hein A, et al. Risk, Prediction and Prevention of Hereditary Breast Cancer - Large-Scale Genomic Studies in Times of Big and Smart Data. Geburtshilfe und Frauenheilkunde. 2018;78(5):481-92.
20. Slavin TP, Maxwell KN, Lilyquist J, Vijai J, Neuhausen SL, Hart SN, et al. The contribution of pathogenic variants in breast cancer susceptibility genes to familial breast cancer risk. NPJ breast cancer. 2017;3:22.
21. Apostolou P, Fostira F. Hereditary breast cancer: the era of new susceptibility genes. BioMed research international. 2013;2013.
22. Ghoussaini M, Pharoah PD. Polygenic susceptibility to breast cancer: current state-of-the-art. Future oncology. 2009;5(5):689-701.
23. Mavaddat N, Pharoah PD, Michailidou K, Tyrer J, Brook MN, Bolla MK, et al. Prediction of breast cancer risk based on profiling with common genetic variants. J Natl Cancer Inst. 2015;107(5).
24. Lewis CM, Vassos E. Prospects for using risk scores in polygenic medicine. Genome medicine. 2017;9(1):96.
25. Pashayan N, Morris S, Gilbert FJ, Pharoah PD. Cost-effectiveness and benefit-to-harm ratio of risk-stratified screening for breast cancer: a life-table model. JAMA oncology. 2018;4(11):1504-10.
26. Maas P, Barrdahl M, Joshi AD, Auer PL, Gaudet MM, Milne RL, et al. Breast Cancer Risk From Modifiable and Nonmodifiable Risk Factors Among White Women in the United States. JAMA oncology. 2016;2(10):1295-302.
27. Owens DK, Davidson KW, Krist AH, Barry MJ, Cabana M, Caughey AB, et al. Medication Use to Reduce Risk of Breast Cancer: US Preventive Services Task Force Recommendation Statement. Jama. 2019;322(9):857-67.
28. Petrucelli N, Daly MB, Pal T. BRCA1- and BRCA2-Associated Hereditary Breast and Ovarian Cancer. GeneReviews® [Internet]. 2016. Available from: https://www.ncbi.nlm.nih.gov/sites/books/NBK1247/.
29. Mavaddat N, Michailidou K, Dennis J, Lush M, Fachal L, Lee A, et al. Polygenic Risk Scores for Prediction of Breast Cancer and Breast Cancer Subtypes. American journal of human genetics. 2019;104(1):21-34.
30. Michailidou K, Lindstrom S, Dennis J, Beesley J, Hui S, Kar S, et al. Association analysis identifies 65 new breast cancer risk loci. Nature. 2017;551(7678):92-4.
31. Sieh W, Rothstein JH, McGuire V, Whittemore AS. The role of genome sequencing in personalized breast cancer prevention. Cancer Epidemiol Biomarkers Prev. 2014;23(11):2322-7.
32. Hughes E, Judkins T, Wagner S, Wenstrup RJ, Lanchbury JS, Gutin A. Development and validation of a residual risk score to predict breast cancer risk in unaffected women negative for mutations on a multi-gene hereditary cancer panel. Journal of Clinical Oncology. 2017;35(15_suppl):1579-.
33. Khera AV, Chaffin M, Aragam KG, Haas ME, Roselli C, Choi SH, et al. Genome-wide polygenic scores for common diseases identify individuals with risk equivalent to monogenic mutations. Nature genetics. 2018;50(9):1219-24.
34. Gail MH, Brinton LA, Byar DP, Corle DK, Green SB, Schairer C, et al. Projecting individualized probabilities of developing breast cancer for white females who are being examined annually. J Natl Cancer Inst. 1989;81(24):1879-86.
35. Costantino JP, Gail MH, Pee D, Anderson S, Redmond CK, Benichou J, et al. Validation studies for models projecting the risk of invasive and total breast cancer incidence. Journal of the National Cancer Institute. 1999;91(18):1541-8.
36. Claus EB, Risch N, Thompson WD. Autosomal dominant inheritance of early-onset breast cancer. Implications for risk prediction. Cancer. 1994;73(3):643-51.
37. Antoniou A, Pharoah PD, Narod S, Risch HA, Eyfjord JE, Hopper JL, et al. Average risks of breast and ovarian cancer associated with BRCA1 or BRCA2 mutations detected in case Series unselected for family history: a combined analysis of 22 studies. Am J Hum Genet. 2003;72(5):1117-30.
38. Claus EB, Risch N, Thompson WD. Genetic analysis of breast cancer in the cancer and steroid hormone study. Am J Hum Genet. 1991;48(2):232-42.

39. Euhus DM, Smith KC, Robinson L, Stucky A, Olopade OI, Cummings S, et al. Pretest prediction of BRCA1 or BRCA2 mutation by risk counselors and the computer model BRCAPRO. J Natl Cancer Inst. 2002;94(11):844-51.
40. Tice JA, Bissell MCS, Miglioretti DL, Gard CC, Rauscher GH, Dabbous FM, et al. Validation of the breast cancer surveillance consortium model of breast cancer risk. Breast cancer research and treatment. 2019;175(2):519-23.
41. Antoniou AC, Pharoah PP, Smith P, Easton DF. The BOADICEA model of genetic susceptibility to breast and ovarian cancer. Br J Cancer. 2004;91(8):1580-90.
42. Lee AJ, Cunningham AP, Kuchenbaecker KB, Mavaddat N, Easton DF, Antoniou AC. BOADICEA breast cancer risk prediction model: updates to cancer incidences, tumour pathology and web interface. British journal of cancer. 2014;110(2):535-45.
43. Amir E, Freedman OC, Seruga B, Evans DG. Assessing women at high risk of breast cancer: a review of risk assessment models. J Natl Cancer Inst. 2010;102(10):680-91.
44. Tyrer J, Duffy SW, Cuzick J. A breast cancer prediction model incorporating familial and personal risk factors. Stat Med. 2004;23(7):1111-30.
45. Maas P, Barrdahl M, Joshi AD, Auer PL, Gaudet MM, Milne RL, et al. Breast Cancer Risk From Modifiable and Nonmodifiable Risk Factors Among White Women in the United States. JAMA oncology. 2016.
46. Easton DF, Pharoah PD, Antoniou AC, Tischkowitz M, Tavtigian SV, Nathanson KL, et al. Gene-panel sequencing and the prediction of breast-cancer risk. The New England journal of medicine. 2015;372(23):2243-57.
47. Shieh Y, Eklund M, Madlensky L, Sawyer SD, Thompson CK, Stover Fiscalini A, et al. Breast Cancer Screening in the Precision Medicine Era: Risk-Based Screening in a Population-Based Trial. J Natl Cancer Inst. 2017;109(5).
48. Familial Breast Cancer: Classification and Care of People at Risk of Familial Breast Cancer and Management of Breast Cancer and Related Risks in People with a Family History of Breast Cancer. 2017 [11.06.2017] Jun; NICE Clinical guideline [CG164]. Available from: https://www.nice.org.uk/guidance/cg164/chapter/recommendations#breast-cancer-risk-category.
49. NCCN Clinical Practice Guidelines in Oncology: Genetic/Familial High-Risk Assessment: Breast, Ovarian, and Pancreatic [Available from: https://www.nccn.org/professionals/physician_gls/pdf/genetics_bop.pdf.
50. Pashayan N, Duffy SW, Chowdhury S, Dent T, Burton H, Neal DE, et al. Polygenic susceptibility to prostate and breast cancer: implications for personalised screening. Br J Cancer. 2011;104(10):1656-63.
51. Muhlberger N, Sroczynski G, Gogollari A, Jahn B, Pashayan N, Steyerberg E, et al. Cost effectiveness of breast cancer screening and prevention: a systematic review with a focus on risk-adapted strategies. Eur J Health Econ. 2021;22(8):1311-44.
